# Photo-Ozonation of Multiclass Pharmaceuticals in Model Water: Kinetic Comparison of UV-C, O_3_ and UV/O_3_ Under Selected pH Conditions

**DOI:** 10.3390/molecules31111930

**Published:** 2026-06-03

**Authors:** Klaudia Całus-Makowska, Anna Grosser, Hanna Białek

**Affiliations:** Faculty of Infrastructure and Environment, Czestochowa University of Technology, J.H. Dąbrowskiego 69, 42-200 Częstochowa, Poland

**Keywords:** pharmaceutical degradation, degradation kinetics, pH effect, UV-C, ozonation, photo-ozonation, advanced oxidation processes, pseudo-first-order kinetics, UV/O_3_

## Abstract

The removal of four representative pharmaceuticals—sulfamethoxazole (SMX), carbamazepine (CBZ), diclofenac (DCF) and ibuprofen (IBU)—was investigated in a model aqueous solution using UV-C photolysis, ozonation and a hybrid UV/O_3_ process. UV-C and UV/O_3_ experiments were conducted at initial pH 3, ~6 and 8, whereas single ozonation was applied at pH ~6 as a near-neutral reference. The processes were compared in terms of removal efficiency, apparent pseudo-first-order kinetics and kinetic enhancement. UV-C photolysis showed pronounced compound selectivity, with efficient removal of DCF and SMX but limited transformation of CBZ and IBU. Ozonation markedly improved the removal of ozone-reactive compounds, particularly CBZ and DCF. Under near-neutral conditions, UV/O_3_ provided high removal efficiencies for all target compounds after 30 min. Kinetic analysis showed that UV/O_3_ enhancement was compound-specific: apparent synergy was observed for CBZ and IBU, with SF values of 1.43 and 1.24, respectively, whereas SMX showed a subadditive response. DCF was rapidly removed under UV/O_3_ but was excluded from the main SF comparison because its concentration approached the lowest calibrated concentration level. These results indicate that UV/O_3_ is especially useful for poorly UV-susceptible pharmaceuticals.

## 1. Introduction

Pharmaceuticals are widely recognised as contaminants of emerging concern (CECs) in the aquatic environment [1]. They are continuously introduced into surface waters, groundwater and, in some cases, drinking water sources, predominantly via incomplete removal in municipal wastewater treatment plants (WWTPs), hospital and household effluents, and, to a lesser extent, pharmaceutical manufacturing and diffuse inputs such as agricultural runoff. Among the numerous pharmaceutically active compounds detected, diclofenac (DCF) [2], ibuprofen (IBU) [3], sulfamethoxazole (SMX) [4] and carbamazepine (CBZ) [5] rank among the most frequently reported in WWTP influents and effluents, surface waters and even groundwater, typically at ng L^−1^–µg L^−1^ levels [6]. Owing to its persistence and limited biodegradability, CBZ is often used as a conservative marker of wastewater-derived contamination. In contrast, DCF and IBU, two widely used non-steroidal anti-inflammatory drugs, and SMX, a sulfonamide antibiotic, represent high-consumption pharmaceuticals frequently detected in aquatic environments and associated with documented ecotoxicological effects.

Conventional WWTPs were not designed to target trace, polar and often recalcitrant pharmaceuticals [7]. Their removal efficiencies are therefore highly compound-dependent and may vary widely with operating conditions and influent composition [8]. While biological processes can partially degrade or transform some pharmaceuticals, many parent compounds and transformation products pass through secondary treatment largely unaffected, resulting in continuous discharge and chronic low-dose exposure of aquatic ecosystems [9,10]. Such persistent exposure, together with evidence of sub-lethal effects on aquatic organisms and the potential contribution to antimicrobial resistance, has prompted the development and implementation of advanced treatment technologies for pharmaceutical abatement.

Among tertiary and quaternary treatment options, advanced oxidation processes (AOPs) have emerged as particularly attractive for the degradation of pharmaceuticals and other CECs [11,12]. AOPs are characterised by the in situ generation of highly reactive oxygen species, principally hydroxyl radicals (•OH), with redox potentials sufficient to oxidise a broad spectrum of organic contaminants, often to more biodegradable intermediates or, in favourable cases, to complete mineralisation. Within this broad class, ozonation (O_3_), UV-based processes and their combination (UV/O_3_) are among the most technologically mature and widely implemented in full-scale water and wastewater treatment.

Direct UV-C irradiation (~254 nm) can photolyse selected pharmaceuticals through excitation of their chromophores, leading to bond cleavage, isomerisation and subsequent reaction with dissolved oxygen. The efficiency of direct photolysis depends strongly on the contaminant’s molar absorption at the irradiation wavelength and its quantum yield; DCF and SMX have been reported as relatively UV-labile, whereas CBZ is comparatively photostable and often only partially removed under similar conditions. Consequently, UV-C photolysis alone typically exhibits strongly compound-specific performance, and in real waters its efficacy may be limited by inner-filter effects and competing absorbance by natural organic matter [13,14].

Ozonation combines the selective reactivity of molecular ozone with the non-selective oxidation capacity of •OH radicals generated via ozone decomposition [15]. Many pharmaceuticals react rapidly with O_3_ through electrophilic attack on electron-rich moieties such as activated aromatic rings, olefinic double bonds, and amine groups, while less ozone-reactive compounds can still be degraded predominantly via •OH. Numerous studies have documented efficient ozonation of CBZ, DCF, SMX and related compounds in both model and real waters, although the observed kinetics and transformation pathways are strongly influenced by molecular structure and water matrix composition [16,17].

The UV/O_3_ hybrid process is designed to exploit potential synergy between these two technologies. UV-C photons can enable direct photolysis of UV-absorbing contaminants and, more importantly, accelerate the photolytic decomposition of dissolved ozone, promoting the formation of hydrogen peroxide and a subsequent radical chain reaction that enhances •OH generation. In UV/O_3_ systems, ozone, •OH and other secondary oxidants can act in parallel, often yielding faster degradation and more extensive transformation than either process alone. In some cases, the observed degradation rates under UV/O_3_ exceed the sum of the individual contributions, suggesting apparent kinetic synergy [18,19]. Recent studies on hybrid oxidation systems and radical-mediated catalytic/PMS-based AOPs have similarly demonstrated that combining multiple oxidative pathways may intensify reactive oxygen species generation and improve degradation performance compared with individual treatment processes [20,21,22,23].

A critical operational parameter for all these AOPs is the solution pH. It governs the speciation and ionisation state of the pharmaceuticals and thus their reactivity and UV absorption, the rate of ozone decomposition and radical formation, and the distribution and lifetime of reactive oxygen species such as O_3_, •OH and secondary radicals (e.g., HO_2_•/O_2_•^−^). For ozonation, increasing pH generally accelerates ozone decomposition, enhancing •OH yields and shifting the process from predominantly selective molecular ozone reactions towards more non-selective radical pathways [24]. In UV/O_3_ systems, pH controls both ozone stability and the speciation of intermediates, with pronounced effects on degradation kinetics and mineralisation efficiency. Even for direct UV photolysis, pH can modulate molar absorption and quantum yields via acid–base equilibria of the target molecules.

Despite a substantial body of work on AOP treatment of pharmaceuticals, several important gaps remain. Many mechanistic and kinetic studies focus on single-compound systems or fixed pH conditions, whereas pharmaceuticals in treated waters typically occur as complex mixtures that may compete for photons, molecular ozone, and reactive radical species. Furthermore, most previous UV/O_3_ investigations have focused on selected pharmaceuticals or specific compound groups, while comprehensive side-by-side comparisons of UV-C, O_3_ and UV/O_3_ processes under harmonised experimental conditions for multiclass pharmaceutical mixtures are still relatively scarce, particularly with respect to pH-dependent behaviour. The present work was conducted in a model aqueous solution to isolate compound-specific and process-specific effects under controlled conditions; therefore, the results should be interpreted as a kinetic benchmark rather than as direct validation in real wastewater matrices. In addition, the pharmaceutical concentrations applied in the present study were intentionally selected at concentrations higher than those typically detected in environmental waters and wastewater to ensure reliable analytical quantification and robust comparative kinetic evaluation under controlled laboratory conditions.

The four pharmaceuticals selected in this work, DCF, IBU, SMX and CBZ, are representative and complementary from both environmental and mechanistic viewpoints. They cover different therapeutic classes, including non-steroidal anti-inflammatory drugs, sulfonamide antibiotics, and antiepileptics, possess distinct pK_a_ values, chromophores, and functional groups, and are expected to exhibit contrasting behaviour toward direct photolysis, ozone, and •OH attack. Their high consumption, frequent detection in municipal effluents and receiving waters, persistence and potential ecotoxicity render them a relevant model mixture for benchmarking AOP performance.

Against this background, the present study compares UV-C irradiation, single ozonation (O_3_), and the hybrid UV/O_3_ process for the degradation of a four-component pharmaceutical mixture containing DCF, IBU, SMX, and CBZ. UV-C and UV/O_3_ were investigated at initial pH values of 3, ~6, and 8, whereas single ozonation was performed at an initial pH of ~6 as a near-neutral reference process. This condition was selected because ozonation chemistry is strongly pH-dependent: under mildly acidic to near-neutral conditions, direct reactions with molecular ozone may contribute substantially, whereas increasing pH promotes ozone decomposition and enhances indirect hydroxyl-radical-mediated oxidation [17,25]. Thus, ozonation at pH ~6 provided a benchmark for comparing molecular-ozone-driven oxidation with UV-assisted ozone activation, without extending the study to a full pH optimisation of ozonation alone.

## 2. Results and Discussion

### 2.1. Performance of Individual Processes: UV-C Photolysis and Ozonation

To establish a baseline for evaluating the hybrid system, the degradation of the pharmaceutical mixture was first examined under the individual action of UV-C irradiation and ozonation. Assessing these single processes under harmonised conditions allows differentiation between compound-specific reactivity and process-driven effects. This provides a reference framework for interpreting the performance of the combined UV/O_3_ process discussed later and for differentiating between additive and potentially enhanced oxidation behaviour. It should also be emphasised that the pharmaceutical concentrations used in the present study were higher than those typically encountered in environmental waters. Under environmentally relevant conditions, oxidation kinetics and process efficiency may be additionally influenced by lower target-compound concentrations, natural organic matter, inorganic radical scavengers and competition for reactive oxygen species in complex wastewater matrices.

**Figure 1:**
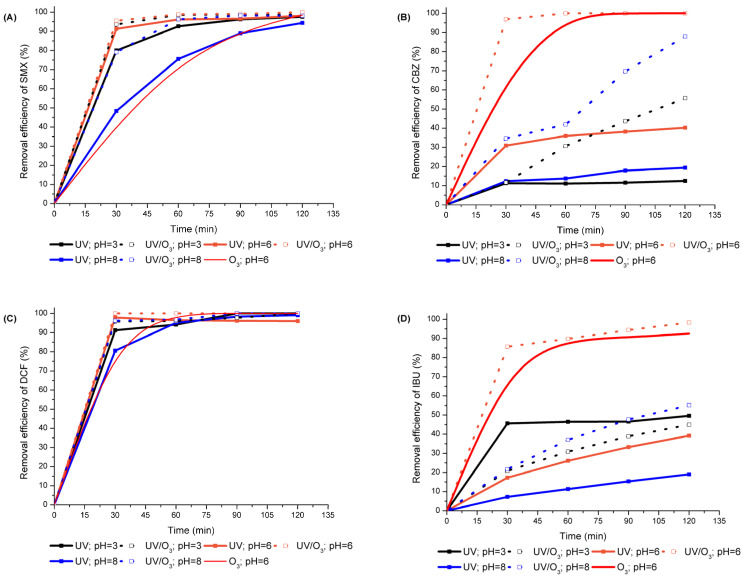
Removal efficiency of (**A**) SMX, (**B**) CBZ, (**C**) DCF and (**D**) IBU during UV-C photolysis, ozonation and UV/O_3_ treatment. UV-C and UV/O_3_ were evaluated at pH 3, ~6 and 8, whereas ozonation was performed only at pH ~6 as a near-neutral reference process. Values approaching 100% indicate apparent parent-compound removal based on HPLC-DAD quantification and should not be interpreted as mineralisation.

The degradation behaviour of DCF, IBU, SMX and CBZ under UV-C irradiation, ozonation and hybrid UV/O_3_ treatment is presented in Figure 1, whereas the complete removal-efficiency dataset for all tested compounds, processes, pH values and sampling times is provided in Table S1. The three AOP configurations exhibited distinct removal patterns, reflecting differences in compound-specific reactivity and process-dependent oxidation pathways.

#### 2.1.1. UV Photolysis

Under direct UV-C irradiation (254 nm), REs varied substantially among the pharmaceuticals and were strongly dependent on molecular structure. DCF and SMX showed the highest susceptibility to photolysis, with rapid decreases in C/C_0_ observed over the irradiation period, whereas CBZ and, particularly, IBU were degraded more slowly.

The pronounced susceptibility of DCF to UV-C photolysis observed in this study is consistent with its molecular structure, which contains two chlorinated aromatic rings and an anilide moiety forming an extended conjugated chromophore that strongly absorbs at 254 nm. As a result, DCF undergoes efficient direct photolysis via excitation of the aromatic system, followed by dechlorination, decarboxylation and hydroxylation reactions, leading to rapid concentration decline [2]. In the present mixture, DCF removal under UV-C exceeded 98% at pH 6 after 30 min and approached apparent complete parent-compound removal within 60–90 min depending on pH, indicating very high intrinsic photoreactivity. Comparable UV-C studies typically report DCF removals of ~60–80% after 60 min of irradiation in aqueous systems, depending on matrix composition and operating conditions [13,26]. The faster decay observed here, therefore, falls at the upper end of the reported kinetic range and confirms that DCF remains highly photolabile even in the presence of co-occurring pharmaceuticals. Similar rapid photodegradation has been reported in UV-C treatment of aqueous DCF, where 83.8% removal was achieved after 10 min and 88.5% after 15 min of irradiation [27], closely matching the near-complete degradation observed in the present system.

Similarly to DCF, SMX demonstrated high susceptibility to direct UV-C photolysis, although its degradation kinetics were more strongly influenced by solution pH. After 30 min of irradiation, SMX removal reached 79.9% at pH 3 and 91.3% at pH 6, whereas only 48.4% degradation was observed at pH 8, indicating reduced photolytic efficiency under alkaline conditions. The degradation progressed steadily with irradiation time, achieving 92.6–96.1% removal at 60 min and high apparent parent-compound removal after 120 min across all tested pH values (94.4–98.2%). This behaviour is consistent with the known photoreactivity of SMX, which contains a UV-absorbing aromatic sulfonamide chromophore enabling efficient photon absorption at 254 nm. A high reactivity of SMX under 254 nm irradiation has been consistently reported. Rapid SMX photodegradation under UV-C light with pseudo-first-order kinetics and a strong dependence on solution pH was demonstrated in controlled aqueous systems [28]. Similarly, efficient direct UV-C removal of SMX, associated with its aromatic sulfonamide and isoxazole chromophores, has been observed in photolysis studies and UV-based advanced oxidation processes [29].

In contrast to DCF and SMX, both CBZ and IBU exhibited substantially lower susceptibility to direct UV-C photolysis, confirming their relative persistence under irradiation at 254 nm. CBZ removal remained limited to 11–40% after 120 min, depending on pH, while IBU degradation did not exceed ~50% even under the most favourable conditions (pH 3). Such behaviour is consistent with reports in the literature describing CBZ as poorly degraded by direct UV-C photolysis and IBU as a compound with low photon absorption and slow phototransformation kinetics at 254 nm [30,31]. Given the limited effectiveness of direct UV-C photolysis for CBZ and IBU, ozonation was subsequently evaluated as an alternative single oxidation process to assess the intrinsic reactivity of the studied pharmaceuticals toward molecular ozone prior to examining the hybrid UV/O_3_ system.

#### 2.1.2. Ozonation

In contrast to direct UV-C photolysis, ozonation provided markedly higher degradation efficiencies for all studied pharmaceuticals, particularly for the more UV-resistant compounds CBZ and IBU, confirming the high oxidative capacity of molecular ozone. Rapid and extensive removal was observed under ozonation alone at neutral pH, with apparent complete parent-compound removal of CBZ and DCF achieved within 60 min, while SMX and IBU reached 98.5% and 92.6% removal after 120 min, respectively. Notably, CBZ, which exhibited strong resistance to UV-C irradiation, was almost completely eliminated by ozonation (>99% at 60 min), indicating a fundamentally different reactivity pattern toward ozone compared to direct photolysis.

The results indicate a strong dependence of removal efficiency on the molecular structure of the pollutants and their specific reactivity towards molecular ozone (O_3_) and hydroxyl radicals (•OH). Among the tested compounds, DCF and CBZ exhibited the highest susceptibility to ozone oxidation. DCF removal reached 87.3% within the first 30 min, achieving complete elimination (100%) shortly thereafter. Similarly, CBZ showed rapid degradation, increasing from 67.2% at 30 min to >99% at 60 min. This behaviour is consistent with the electrophilic nature of ozone, which selectively attacks electron-rich moieties. In the case of CBZ, the reaction proceeds primarily via the 1,3-dipolar cycloaddition of ozone to the olefinic double bond in the dibenzazepine ring, following the Criegee mechanism [32,33]. For DCF, the high reactivity is attributed to the presence of the secondary amine group and the aromatic rings activated by electron-donating substituents, facilitating rapid direct ozone attack even at slightly acidic pH [34,35]. SMX displayed a distinct kinetic profile, characterised by a slower initial reaction rate (41.3% removal at 30 min) compared to DCF and CBZ, but eventually reaching high removal efficiency (98.5%) after 120 min.

The reaction of SMX with ozone typically involves electrophilic attack on the amine group of the aniline ring or the isoxazole ring. The observed lag phase and lower initial efficiency at pH 6 can be correlated with the speciation of SMX and mass transfer limitations, which have been recently identified as critical factors in SMX ozonation kinetics [36]. As the process proceeds, the generation of intermediates and the potential shift towards radical-driven mechanisms likely contribute to the final high removal rates.

IBU proved to be the most recalcitrant among the studied pharmaceuticals, exhibiting a “plateau” effect. Although 75.9% removal was achieved in the first 30 min, the efficiency increase slowed significantly in subsequent intervals (89.4% at 60 min, reaching only 92.6% at 120 min). IBU lacks strong electron-donating groups capable of reacting directly with O_3_, making its degradation heavily dependent on non-selective •OH radicals generated from ozone decomposition [35]. At pH 6, the decomposition and subsequent radical generation are less favourable than at alkaline conditions, which explains the incomplete removal and the persistence of IBU relative to the other compounds. This limitation highlights the potential benefit of coupling ozonation with catalytic or photolytic processes (e.g., UV/O_3_) to enhance the mineralisation of ozone-resistant micropollutants [37,38]. Additionally, possible pH drift during ozonation and UV/O_3_ treatment was not monitored continuously and may have influenced ozone decomposition kinetics, reactive oxygen species distribution and oxidation pathways during the experiments.

Recent studies emphasise that while parent compound removal is often high, the formation and persistence of potentially toxic transformation products (TPs) remain a concern. For instance, CBZ ozonation yields acridine and epoxide derivatives [33], while IBU degradation can lead to the formation of persistent hydroxylated and decarboxylated by-products [35]. Therefore, the obtained REs should be interpreted primarily as indicators of parent-compound degradation, pointing towards the need for toxicity assessment of the treated effluent [33,37]. Consequently, the environmental implications of the investigated treatments cannot be fully assessed without complementary transformation-product identification and toxicity evaluation.

The ozonation results should be interpreted in relation to both the nominal applied ozone input reported in the methods section and the composition of the model solution. Although the applied ozone input was relatively high when expressed on a liquid-volume basis, the model solution was also characterised by a high initial COD of 1137 mg O_2_ L^−1^, mainly due to methanol introduced with the pharmaceutical stock solution. Therefore, normalisation of the ozone input to organic load provides a more appropriate basis for comparison with literature data than the volumetric ozone input alone. In the present study, the cumulative ozone input corresponded to approximately 0.17–0.69 mg O_3_ mg^−1^ COD over 30–120 min.

The literature data show that ozone requirements for pharmaceutical abatement vary substantially depending on matrix composition, background organic load and compound-specific reactivity. In treated municipal effluents, micropollutant removal is often achieved at lower volumetric ozone doses because target compounds occur at trace concentrations and the organic matrix differs from that of concentrated model solutions [39,40]. When the ozone dose is normalised to organic load, for instance, de Wilt et al. (2018) reported complete CBZ removal at approximately 0.4–0.5 g O_3_ g^−1^ TOC in a combined biological–ozonation system [41], while in hospital wastewater, Mansımlı et al. (2025) reported that complete removal of detected pharmaceutically active compounds required approximately 1.5 mg O_3_ mg^−1^ COD, whereas SMX degradation occurred at a considerably lower non-stoichiometric ozone dose of approximately 0.05 mg O_3_ mg^−1^ COD [42].

### 2.2. Performance and Apparent Kinetic Synergy of the UV/O_3_ Process

The UV/O_3_ process was examined as a hybrid oxidation system integrating direct UV-C photolysis with ozone-mediated oxidation. Removal efficiency obtained for UV/O_3_ at pH 3, ~6 and 8 is presented in Figure 1, while the complete numerical dataset for all processes, pH conditions, compounds and sampling times is provided in Table S1. Compared with UV-C photolysis alone, UV/O_3_ substantially improved the removal of the less UV-susceptible compounds, particularly CBZ and IBU. Consequently, the hybrid process provided a more balanced removal profile across the four-component pharmaceutical mixture.

At pH ~6, where direct comparison among UV-C, O_3_ and UV/O_3_ was possible, the hybrid process showed high parent-compound removal already during the initial stage of treatment. After 30 min of UV/O_3_ exposure, removal efficiencies reached approximately 95.5% for SMX, 96.9% for CBZ, 100% for DCF and 85.7% for IBU. This response differed from the more compound-selective behaviour of the individual processes: UV-C photolysis was most effective for SMX and DCF, whereas ozonation was more effective for CBZ and DCF. UV/O_3_ thereby broadened the treatment system’s removal spectrum and improved early-stage removal of compounds that were less efficiently degraded by the single processes.

The enhanced performance of UV/O_3_ is consistent with the established photochemical behaviour of ozone under UV-C irradiation. Ozone absorbs radiation at 254 nm, and its photolysis may initiate reaction sequences leading to the formation of hydrogen peroxide and hydroxyl radicals [43,44,45].

O_3_ + H_2_O + hν → H_2_O_2_ + O_2_ (1)

H_2_O_2_ + hν → 2•OH (2)

2O_3_ + H_2_O_2_ → 2•OH + 3O_2_ (3)

These reactions are presented as commonly proposed pathways for UV/O_3_ systems; however, they were not directly verified in the present study.

Comparable radical-driven degradation behaviour has also been reported in other advanced oxidation and catalytic systems designed for pharmaceutical and organic-contaminant removal, further confirming the importance of reactive oxygen species in determining process efficiency [21,22,23,46]. Therefore, the observed degradation behaviour should be interpreted as being consistent with a potentially greater contribution of radical-mediated oxidation, rather than as direct evidence of •OH radical formation. The potential enhancement of reactive oxygen species formation under UV/O_3_ conditions may shift the oxidation pathway from predominantly selective electrophilic reactions, characteristic of molecular ozone, toward less selective oxidative reactions such as radical abstraction and addition [47]. Under acidic conditions, molecular ozone remains relatively stable and selective oxidation by direct ozone reactions is expected to dominate. As pH increases, ozone decomposition becomes progressively accelerated through hydroxide-ion-initiated chain reactions, promoting the formation of reactive oxygen species, particularly hydroxyl radicals (•OH). In UV/O_3_ systems, UV irradiation may further enhance ozone photolysis and generate secondary reactive oxygen species, thereby shifting the oxidation mechanism toward less selective, radical-mediated pathways. Consequently, pH not only influences ozone stability but also affects the relative contribution of molecular ozone and indirect radical oxidation during pharmaceutical degradation. This effect may be particularly relevant for compounds such as IBU, which lack electron-rich moieties highly susceptible to direct ozone attack. The higher apparent degradation rate of SMX under UV/O_3_ conditions (k_obs_ = 0.052 min^−1^) compared with ozonation alone suggests that UV-assisted ozone activation may have contributed to SMX transformation at pH ~6, although the subadditive SF value suggests that the observed enhancement should be interpreted cautiously.

The statistical comparison of 30 min removal efficiencies confirmed that the differences among UV-C, O_3_ and UV/O_3_ at pH ~6 were significant for all target compounds. One-way ANOVA showed significant treatment effects for SMX (F(2, 6) = 170.47, *p* < 0.001), CBZ (F(2, 6) = 264.66, *p* < 0.001), DCF (F(2, 6) = 9.73, *p* = 0.013) and IBU (F(2, 6) = 368.08, *p* < 0.001).

Tukey’s post hoc comparisons further indicated that UV/O_3_ significantly increased the 30 min removal of CBZ and IBU relative to UV-C photolysis (*p* < 0.001), supporting the interpretation that the hybrid process was particularly beneficial for compounds poorly removed by direct UV-C irradiation (Table S4).

To assess whether the improved removal observed under UV/O_3_ represented a kinetic enhancement beyond the additive contribution of UV-C photolysis and ozonation, apparent pseudo-first-order rate constants were used to calculate the synergy factor at pH ~6. The complete set of apparent kinetic parameters estimated for all pH values, processes and compounds is reported in Table S2, whereas the rate constants used for $S_{F}$ calculation are summarised in Table 1. This analysis was restricted to pH ~6, as this was the only condition for which complete UV-C, O_3_ and UV/O_3_ datasets were available.

The kinetic assessment demonstrated that the benefit of UV/O_3_ was compound-specific and should not be interpreted as uniform synergy for all target pharmaceuticals. Based on the available apparent kinetic constants, apparent kinetic synergy was identified for CBZ and IBU, with SF values of 1.43 and 1.24, respectively (Table 1). For CBZ, this result is particularly relevant because direct UV-C photolysis contributed only marginally to its removal, whereas the UV/O_3_ process yielded a rate constant exceeding the sum of the individual UV-C and O_3_ contributions. For IBU, SF > 1 indicates that the hybrid process enhanced degradation beyond the additive effect of the two single processes, partly compensating for the low efficiency of UV-C photolysis and the slower residual removal observed during ozonation.

By contrast, SMX exhibited a subadditive response, with SF = 0.81, indicating that the apparent UV/O_3_ rate constant did not exceed the sum of the UV-C and O_3_ contributions. The case of DCF requires separate consideration. DCF was rapidly removed under UV/O_3_ conditions and was not included in the main SF comparison because its concentration rapidly approached or decreased below the lowest calibrated concentration level, which limited robust pseudo-first-order fitting based on reliably quantifiable concentration points. Therefore, DCF was excluded from the main SF comparison presented in Table 1.

The regression-slope comparison at pH ~6 further supported the compound-specific character of the kinetic enhancement. Significant treatment-dependent differences in apparent degradation slopes were found for CBZ (F(2, 4) = 24.43, *p* = 0.006) and IBU (F(2, 9) = 10.38, *p* = 0.005), which is consistent with the SF > 1 values obtained for these compounds (Table 1). In contrast, no significant slope differentiation was detected for SMX (F(2, 8) = 1.29, *p* = 0.327), in agreement with its subadditive SF value.

The kinetic constants obtained in the conducted experiments are broadly consistent with literature trends, although direct numerical comparison should be made with caution because apparent k_obs_ values are highly dependent on process configuration, ozone dose, UV-C fluence, initial concentration and matrix composition. Selected literature values reported for the investigated pharmaceuticals are summarised in Table S3.

For DCF, the apparent UV-C rate constants obtained in the present study were of the same order of magnitude as values previously reported for direct UV-based degradation [2,13,26]. The ozonation rate obtained in the present study was also comparable to the lower range of pseudo-first-order constants reported for DCF ozonation in laboratory bubble-column systems, where values of approximately 0.074–0.098 min^−1^ have been reported depending on operating conditions [48]. This agreement supports the interpretation that DCF is highly susceptible to both photolytic and ozone-mediated parent-compound removal.

The kinetic behaviour of CBZ was also consistent with the literature. The low k_obs_ value observed during UV-C photolysis agrees with reports describing CBZ as poorly susceptible to direct photolysis at 254 nm. For example, Im et al. reported a pseudo-first-order degradation rate of approximately 0.29 × 10^−2^ min^−1^ for CBZ photolysis in the presence of methanol, which is close to the low UV-C rate obtained in the present study [49]. Conversely, the substantially higher k_obs_ values obtained under ozonation and UV/O_3_ are consistent with the high reactivity of CBZ toward molecular ozone and the documented formation of ozonation products resulting from attack on the dibenzazepine ring system [17,32,33,50].

For IBU, the low UV-C rate constant determined in the present study is consistent with the literature, indicating that IBU undergoes relatively slow direct photolysis compared with more UV-labile pharmaceuticals [30]. Reported photolytic rate constants for IBU are typically in the low 10^−3^ to 10^−2^ min^−1^ range, depending on the irradiation source, matrix and initial concentration. The higher k_obs_ obtained for UV/O_3_ in the conducted experiments indicates that the hybrid process increased the contribution of oxidative pathways beyond direct photolysis. This interpretation is consistent with reports showing that IBU degradation is improved in oxidation systems that enhance the availability of less-selective reactive oxygen species, although the magnitude of the rate enhancement depends strongly on process configuration [30,35].

For SMX, the rate constants determined in the conducted experiments were lower than some values reported for direct UV-C photodegradation. For instance, Mouamfon et al. reported pseudo-first-order constants ranging from 0.170 to 0.932 min^−1^ for SMX under UV-C irradiation, depending on the initial concentration and experimental conditions [51]. The lower values observed here may be related to the substantially different experimental system, including the multicompound pharmaceutical mixture, high nominal pharmaceutical concentration, and the relatively high initial COD associated with methanol introduced with the stock solution. These factors may increase the overall oxidant demand and influence apparent degradation kinetics, particularly in radical-mediated processes.

### 2.3. Influence of Initial pH on Degradation Kinetics

The influence of initial pH was evaluated for UV-C photolysis and UV/O_3_ treatment, whereas single ozonation was performed only at pH ~6 and was therefore excluded from the pH-dependent comparison. Consequently, the discussion of pH effects refers specifically to UV-based processes. The ozonation data are used only as a near-neutral reference for interpreting the relative contribution of molecular ozone under the conditions applied in this study. This distinction is important because ozonation chemistry is strongly pH-dependent: under mildly acidic to near-neutral conditions, direct reactions with molecular ozone may contribute substantially, whereas increasing pH generally promotes ozone decomposition and enhances the contribution of hydroxyl-radical-mediated oxidation [17,25].

Initial pH affected degradation kinetics in a compound-specific manner. This behaviour can be attributed to the combined influence of pharmaceuticals, changes in UV-C absorption, molecular structure and, in the case of UV/O_3_, pH-dependent ozone decomposition and secondary radical formation. In direct UV-C photolysis, pH mainly influences the ionisation state of the target compounds and may modify their molar absorption and quantum yields. Similar pH-dependent effects have been reported for UV-based degradation of sulfonamide antibiotics and non-steroidal anti-inflammatory drugs, where changes in molecular speciation can alter photolytic susceptibility and transformation pathways [13,28,29].

Under UV-C irradiation, DCF and SMX were the most susceptible compounds across the investigated pH range. DCF showed high apparent removal under acidic, near-neutral and alkaline conditions, indicating that direct photolysis was efficient for this compound irrespective of the initial pH. This behaviour is consistent with previous studies showing that DCF is readily photodegraded under UV-C irradiation due to its aromatic chromophoric structure and susceptibility to photochemical transformations, including dechlorination, decarboxylation, and hydroxylation [2,13,26]. However, values approaching 100% removal should be interpreted as apparent parent-compound removal rather than complete degradation or mineralisation.

SMX also exhibited high susceptibility to UV-C irradiation, but its early-stage removal was more clearly affected by pH. The initial removal after 30 min was higher at pH 3 and ~6 than at pH 8, indicating reduced photolytic efficiency under alkaline conditions. This observation is consistent with reports showing that SMX photodegradation can be strongly influenced by pH-dependent speciation and the associated changes in UV-C absorption and reaction pathways [28,29]. Nevertheless, after prolonged irradiation, high apparent parent-compound removal was observed across all tested pH values, suggesting that the pH effect was most pronounced during the early stages of UV-C treatment.

In contrast, CBZ and IBU remained substantially more resistant to direct UV-C photolysis. CBZ removal was limited at all investigated pH values, confirming that pH adjustment alone did not substantially enhance its photolytic degradation under the applied UV-C conditions. This is consistent with the frequently reported persistence of CBZ during direct UV treatment and its relatively low susceptibility to photolysis at 254 nm [31]. IBU showed its highest UV-C removal under acidic conditions, whereas alkaline pH resulted in particularly low removal. The limited direct photolysis of IBU agrees with previous studies indicating that this compound exhibits relatively low photon absorption and slow phototransformation kinetics under UV-based treatment unless additional oxidants or catalysts are applied [30].

In the UV/O_3_ system, the influence of pH was more complex because degradation may proceed through simultaneous direct photolysis, direct molecular ozone oxidation and secondary radical-mediated pathways. This mechanistic complexity is characteristic of UV/O_3_ systems, where UV-C irradiation promotes ozone activation and may enhance the formation of reactive oxygen species, including hydroxyl radicals [43,44,52]. Near-neutral pH provided the most favourable early-stage removal for CBZ and IBU. This is particularly relevant because both compounds were poorly removed by UV-C alone. For CBZ, UV/O_3_ at pH ~6 resulted in rapid apparent parent-compound removal, whereas acidic and alkaline conditions produced slower removal. This behaviour is consistent with the high reactivity of CBZ toward ozone, especially through attack on the olefinic double bond in the dibenzazepine ring, combined with the additional oxidative contribution expected in photo-ozonation systems [17,32,33].

For IBU, UV/O_3_ also showed markedly higher removal at pH ~6 than at pH 3 or 8, suggesting that near-neutral conditions favoured the combined action of ozone activation and secondary oxidation pathways for this compound. Literature data indicate that IBU is less reactive toward direct molecular ozone than more electron-rich pharmaceuticals and may therefore benefit from processes that increase the contribution of non-selective oxidative species [30,35]. However, this interpretation should remain cautious because neither dissolved ozone exposure nor hydroxyl-radical concentration was directly measured in the present study.

The behaviour of SMX in the UV/O_3_ system was less straightforward. Although high removal was observed under all tested pH conditions, the synergy analysis at pH ~6 indicated a subadditive response. This suggests that direct UV-C photolysis and ozonation already contributed substantially to SMX removal, and their combination did not produce a clear kinetic gain beyond the sum of the individual processes. Such behaviour is plausible because SMX can undergo both direct UV-C transformation and ozone-mediated oxidation, while its apparent degradation kinetics may be affected by pH, molecular speciation and gas–liquid mass-transfer limitations [28,29,36]. Therefore, high removal efficiency in the UV/O_3_ system should not automatically be interpreted as evidence of kinetic synergy.

DCF was rapidly removed under UV/O_3_ conditions across the investigated pH range. This is consistent with its high susceptibility to both UV-C photolysis and ozone-mediated transformation, as reported in previous studies on DCF degradation by UV-based and ozonation processes [2,13,26,35]. However, in the present study, DCF concentrations reached values close to or below the lowest calibrated concentration level at early reaction times, particularly under near-neutral UV/O_3_ conditions. As a result, robust pH-dependent kinetic comparison for DCF is limited by the rapid disappearance of the parent compound. For this reason, DCF should be discussed primarily in terms of rapid apparent parent-compound removal rather than precise kinetic differentiation among pH conditions.

The statistical analysis of 30 min removal efficiencies confirmed that both the treatment process and the initial pH significantly affected the performance of UV-based processes. In the two-way ANOVA comparing UV-C and UV/O_3_ across pH 3, ~6 and 8, the process, pH and process × pH interaction terms were significant for all four pharmaceuticals (Table S5). For SMX, the corresponding values were F(1, 12) = 68.56, *p* < 0.001 for process, F(2, 12) = 84.67, *p* < 0.001 for pH and F(2, 12) = 15.80, *p* < 0.001 for the interaction. For CBZ, the same factors were also highly significant (F(1, 12) = 789.29, F(2, 12) = 909.18, F(2, 12) = 335.61; *p* < 0.001). Significant effects were likewise observed for IBU (F(1, 12) = 379.30, F(2, 12) = 461.27, F(2, 12) = 738.80; *p* < 0.001). For DCF, the process effect (F(1, 12) = 14.06, *p* = 0.003), pH effect (F(2, 12) = 9.39, *p* = 0.004) and interaction (F(2, 12) = 4.33, *p* = 0.038) were significant, although interpretation for this compound should consider its rapid approach to apparent complete parent-compound removal under several treatment conditions.

Tukey’s post hoc comparisons further clarified the compound-specific nature of these effects (Tables S6–S9). For CBZ, UV/O_3_ at pH ~6 produced significantly higher removal than all other UV-based conditions, including UV-C at pH ~6 and UV/O_3_ at pH 3 and 8 (*p* < 0.001; Table S7). A similarly strong response was observed for IBU, for which UV/O_3_ at pH ~6 significantly outperformed UV-C at all tested pH values and UV/O_3_ at pH 3 and 8 (*p* < 0.001; Table S9). These results indicate that near-neutral photo-ozonation was particularly favourable for compounds with low susceptibility to direct UV-C photolysis. For SMX, the post hoc analysis showed that UV/O_3_ at pH 3 and ~6 was significantly more effective than UV-C at pH 8, whereas the difference between UV-C and UV/O_3_ at pH ~6 was not significant (*p* = 0.809; Table S6). This reflects the already high UV-C removal of SMX under near-neutral conditions. For DCF, most comparisons among UV/O_3_ conditions were not significant, while significant contrasts were mainly associated with the lower removal observed under UV-C at pH 8 (Table S8). The post hoc comparisons, therefore, indicate that the pH-dependent benefit of UV/O_3_ was most pronounced for CBZ and IBU, particularly under near-neutral conditions. For SMX and DCF, interpretation of pairwise differences is less straightforward because high apparent removal was already achieved under selected UV-C or UV/O_3_ conditions.

### 2.4. Integrated Comparative Assessment of Treatment Performance

**Figure 2:**
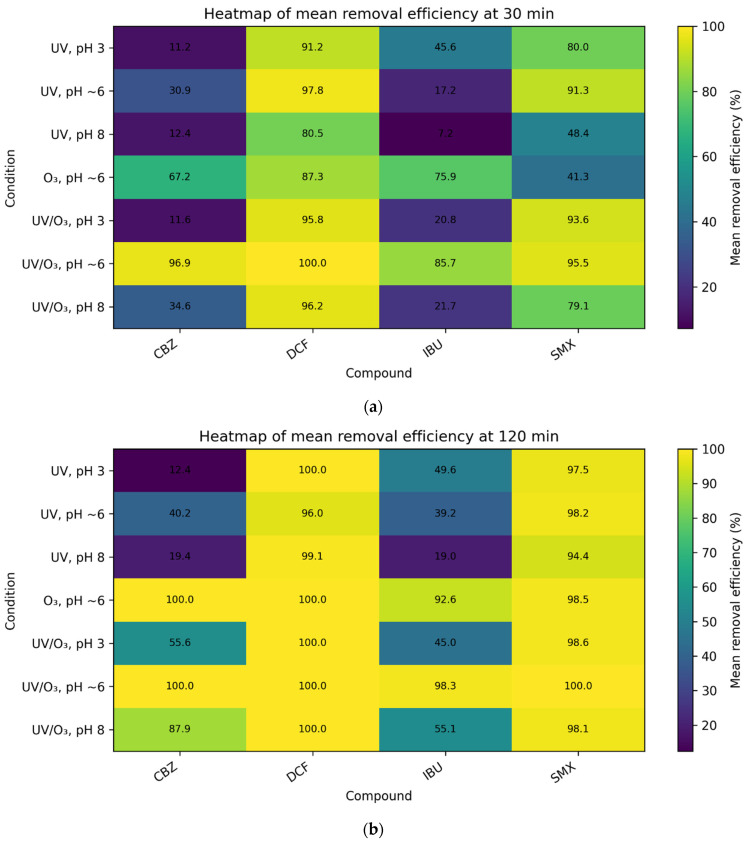
Heatmaps of mean removal efficiency for SMX, CBZ, DCF and IBU after (**a**) 30 min and (**b**) 120 min of treatment under UV-C, O_3_ and UV/O_3_ conditions. UV-C and UV/O_3_ were evaluated at pH 3, ~6 and 8, whereas ozonation was performed only at pH ~6 as a near-neutral reference process. Values approaching 100% indicate apparent parent-compound removal based on HPLC-DAD quantification and should not be interpreted as mineralisation.

To synthesise the removal patterns observed across compounds, processes and pH conditions, mean removal efficiencies after 30 and 120 min were visualised as heatmaps (Figure 2a,b). The 30 min time point was selected to represent the early stage of treatment, where differences in compound reactivity and process performance remained clearly distinguishable. In contrast, the 120 min heatmap illustrates the final extent of apparent parent-compound removal under the applied experimental conditions. The complete numerical dataset is provided in Table S1.

The 30 min heatmap confirms the strong compound- and process-dependent character of pharmaceutical removal. UV-C photolysis was highly effective for SMX and DCF under selected pH conditions, but provided limited removal of CBZ and IBU. Ozonation at pH ~6 showed a different response, with high early-stage removal of ozone-reactive compounds, particularly CBZ and DCF. The UV/O_3_ process at pH ~6 provided the most balanced early-stage performance across the four-component mixture, with high removal efficiencies for all target compounds after 30 min. This visual pattern is consistent with the statistical analysis and kinetic assessment, which identified the clearest practical benefit of UV/O_3_ for CBZ and IBU.

The 120 min heatmap shows that prolonged treatment resulted in high apparent removal for most compound-process combinations, particularly for DCF and SMX. However, interpretation of this time point is less discriminating because several values approached apparent complete parent-compound removal. Therefore, the 30 min heatmap is more informative for comparing initial process efficiency, whereas the 120 min heatmap primarily illustrates the final treatment extent.

## 3. Materials and Methods

### 3.1. Chemicals and Reagents

Diclofenac sodium salt (≥98% purity, TLC-verified), ibuprofen sodium salt (≥98% purity, GC-verified), and sulfamethoxazole (GC/HPLC-checked) were purchased from Sigma-Aldrich (Burlington, MA, USA). Carbamazepine (≥97.0% purity) was obtained from Glentham Life Sciences (Corsham, UK). HPLC-grade methanol (≥99.9%), acetonitrile (≥99.9%), ultrapure water, phosphoric acid (H_3_PO_4_), hydrochloric acid (HCl) and sodium hydroxide (NaOH) were from Sigma-Aldrich. HCl and NaOH solutions were used to adjust pH prior to the experiments. A mixed stock solution (1 mg mL^−1^ per compound) was stored at 2–6 °C. For the experiments, a working solution was prepared by 10-fold dilution with ultrapure water to a nominal concentration of 100 mg L^−1^ for each compound; the model solution pH was 5.24. All experiments were conducted under standard laboratory conditions (20–25 °C; atmospheric pressure).

### 3.2. Experiments and Procedure

#### 3.2.1. UV Irradiation (Photolysis)

UV-C photolysis experiments were performed using a Laboratory UV Reactor System 3, UV-RS-3 (Heraeus, Hanau, Germany) equipped with a low-pressure mercury immersion lamp (TNN 15/32; principal emission ~254 nm), an immersion quartz sleeve, and a jacketed reactor vessel. According to the manufacturer’s technical specification, the lamp’s electrical power is 15 W, the total immersion length is 370 mm, the effective arc length is 170 mm, and the optical path in the standard reactor configuration is <2 cm. The working volume was 800 mL. The solution was magnetically stirred throughout each experiment to ensure homogeneous irradiation and mass transfer. Before irradiation, the reaction mixture was equilibrated under stirring, and the t = 0 min aliquot was collected immediately before switching on the UV lamp. Subsequent samples were collected after 30, 60, 90 and 120 min using syringes and filtered through 0.45 µm cellulose acetate membrane filters prior to HPLC-DAD analysis. The same filter type was used for all samples to ensure comparability of the concentration–time profiles. Initial pH was adjusted to 3, ~6, and 8 to assess pH effects; unless otherwise stated, all reported pH values correspond to the initial conditions established before the start of each experiment. Continuous pH monitoring during the oxidation processes was not performed.

#### 3.2.2. Ozonation and UV/O_3_

Batch ozonation and UV/O_3_ experiments were conducted at a working volume of 800 mL, using the same sampling schedule and sample-preparation procedure as in the UV-C tests. Ozonation was performed only at an initial pH of ~6; therefore, these data were used as a near-neutral benchmark rather than as a pH-dependent ozonation study. UV/O_3_ experiments were performed at initial pH values of 3, ~6, and 8 to evaluate pH-dependent photo-ozonation performance.

Ozone was generated from air using a laboratory-scale ozone generator. The gas flow rate was adjusted to 5.0 L min^−1^ and measured with a rotameter. The ozone-containing gas was introduced into the reaction solution through a porous air stone diffuser placed near the bottom of the reactor. The ozone generator output was determined iodometrically by absorbing ozone in potassium iodide solution, followed by titration of the liberated iodine with standardised sodium thiosulfate [53,54]. The measured ozone production rate was 312 ± 12 mg O_3_ h^−1^. This value represents the nominal gaseous ozone input delivered to the reactor and should not be interpreted as dissolved ozone exposure, because dissolved ozone concentration, ozone transfer efficiency and off-gas ozone losses were not determined.

Based on the working volume of 0.8 L, the applied ozone input corresponded to 390 ± 15 mg O_3_ L^−1^ h^−1^, equivalent to 6.5 ± 0.25 mg O_3_ L^−1^ min^−1^. Accordingly, the cumulative applied ozone input was approximately 195 ± 7.5, 390 ± 15, 585 ± 22.5 and 780 ± 30 mg O_3_ L^−1^ after 30, 60, 90 and 120 min, respectively.

The initial chemical oxygen demand (COD) of the model solution was 1137 mg O_2_ L^−1^. This relatively high COD value was mainly due to methanol introduced during the preparation of the pharmaceutical stock solution used to dissolve the target compounds. Therefore, COD was reported only as a matrix-characterisation parameter and was not used as a direct indicator of pharmaceutical mineralisation.

**Figure 3:**
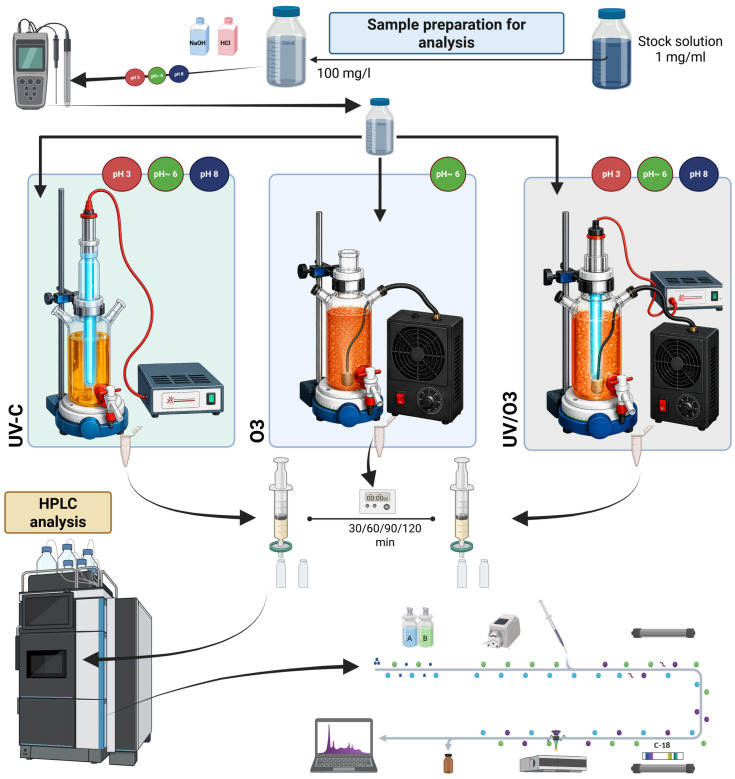
Scheme of experiment.

The experimental setup is shown in Figure 3.

### 3.3. Chromatographic Analysis

#### 3.3.1. Instrumentation

Pharmaceuticals were quantified by HPLC–DAD using a reversed-phase Accucore™ C18 column (2.6 µm, 150 mm × 3 mm i.d.; Thermo Fisher Scientific, Waltham, MA, USA) coupled to a Thermo Scientific UV–VIS diode-array detector (Waltham, MA, USA); data acquisition and processing were performed in Chromeleon™ 7 CDS software (Thermo Fisher Scientific, Waltham, MA, USA).

#### 3.3.2. Chromatographic Conditions

The method followed our previous protocol, adapted from Gilart [55,56], using A = ultrapure water acidified with H_3_PO_4_ to pH 2.8 and B = acetonitrile; the column was held at 40 °C and eluted at 0.60 mL min^−1^ with DAD detection at 230 nm. Total run time: 21 min. The gradient programme was: 0–2.0 min, 90% A (10% B); 2.0–19.0 min, 10% A (90% B); 19.0–21.0 min, 90% A (10% B) re-equilibration.

Before chromatographic analysis, all collected samples were filtered through 0.45 µm cellulose acetate membrane filters. Quantification of SMX, CBZ, DCF and IBU was performed by external calibration based on peak-area integration. A linear calibration model with offset was applied for each compound. The calibration curves showed excellent linearity within the applied working range, with coefficients of determination between 0.99998 and 1.00000. The lowest calibrated concentration level was 1.00 µg mL^−1^ and was treated as the practical lower limit of the calibrated quantification range under the applied HPLC-DAD conditions. Concentrations calculated below this level were considered outside the calibrated quantification range and were interpreted cautiously, particularly in kinetic modelling. Consequently, kinetic parameters obtained for datasets approaching this concentration range should be regarded as apparent, process-specific estimates rather than intrinsic degradation constants.

### 3.4. Data Analysis

Concentration–time data for CBZ, DCF, IBU and SMX were processed as C/C_0_ (dimensionless), where *C* is the measured concentration at time *t* and *C*_0_ is the initial concentration at *t* = 0. Removal efficiency (RE, %) at each sampling point was calculated as

(4) $$RE=(1-C/C_{0})\times 100$$

Assuming pseudo-first-order (PFO) decay, kinetic parameters were obtained by linear regression of ln(C/C_0_) versus time (min) for each compound under the investigated process (O_3_, UV-C, UV/O_3_) and pH condition (≈3, 6, 8):

(5) $$ln\;(C/C_{0})=-k_{\mathrm{obs}}\;t$$

Yielding the observed rate constant $k_{\mathrm{obs}}$ (min^−1^) and the coefficient of determination (R^2^). Half-life was computed as $t_{1/2}=ln\;(2)/k_{\mathrm{obs}}$. Where at least three positive data points were available (df > 0), 95% confidence intervals (CI) for $k_{\mathrm{obs}}$ were derived from the slope standard error and *t*-distribution. For short series (*n* < 3), $k_{\mathrm{obs}}$ parameters are reported without CI and interpreted cautiously. Kinetic fitting was performed exclusively using concentration data within the validated calibration range. Data points that approached or fell below the lowest calibration level were excluded from the regression analysis to avoid distorting the calculated rate constants. Non-positive C/C_0_ values (from analytical noise at near-complete removals) were excluded from the log-transform only; raw RE values were retained. Particular caution was applied when interpreting pseudo-first-order fits for datasets in which concentrations approached or decreased below the lowest calibrated concentration level.

The presence of methanol originating from the stock solution represents an important limitation of the model system. Methanol may compete with target pharmaceuticals for reactive oxygen species, particularly hydroxyl radicals, and therefore may affect the apparent kinetics of UV/O_3_ and ozonation processes. Consequently, the obtained rate constants should be interpreted as process-specific apparent values for the tested model matrix rather than intrinsic degradation constants.

To evaluate the apparent kinetic synergy of the hybrid UV/O_3_ process, a synergy factor (SF) was calculated using the apparent pseudo-first-order rate constants obtained at pH ~6:

(6) $$SF=\frac{k_{UV/O3}}{k_{UV}+k_{O3}}$$

where k_UV/O3_, k_UV_ and k_O3_ denote the apparent rate constants for UV/O_3_, UV-C photolysis and single ozonation, respectively.

This approach follows the common kinetic rationale used for hybrid oxidation processes, in which the performance of the combined process is compared with the sum of the individual processes to assess whether the combined treatment exceeds an additive effect [52,57,58]. Values of SF > 1 indicate apparent kinetic synergy, SF ≈ 1 indicates an additive effect, and SF < 1 indicates a subadditive response. Because single ozonation was performed only at pH ~6, SF was calculated exclusively at this pH. Accordingly, SF was used as an apparent kinetic indicator of process enhancement, while mechanistic interpretation remains limited by the absence of dissolved ozone exposure, hydroxyl-radical and transformation-product measurements.

Removal-efficiency values are reported as a mean based on three replicate measurements (*n* = 3). Statistical evaluation of removal efficiency was performed using two complementary approaches. For UV-based treatments, the effects of process type, initial pH and their interaction were assessed after 30 min using two-way ANOVA, with process (UV-C, UV/O_3_) and pH (3, ~6, 8) as categorical factors. At pH ~6, where UV-C, O_3_ and UV/O_3_ datasets were all available, differences among the three treatment processes were evaluated using one-way ANOVA. Additionally, Tukey’s post hoc test was used for pairwise comparisons where significant effects were observed. Differences were considered statistically significant at *p* < 0.05. Before performing the ANOVA analysis, its basic assumptions were verified. The normality of the residual distribution was assessed using the Shapiro–Wilk test, while the homogeneity of variances across groups was assessed using Levene’s test.

Differences between apparent pseudo-first-order rate constants at pH ~6 were additionally evaluated by comparing regression slopes of ln(C/C_0_) versus time using linear models with treatment × time interaction terms. This analysis was applied only to datasets with sufficient quantifiable concentration points.

All analyses were performed in STATISTICA and R (v4.2.2) with reproducible scripts (tidy data wrangling, linear models, and plotting).

## 4. Conclusions

This study compared UV-C photolysis, single ozonation and UV/O_3_ treatment for the removal of a four-component pharmaceutical mixture containing SMX, CBZ, DCF and IBU in a model aqueous solution. The results confirmed that the performance of the investigated processes was strongly compound-dependent. UV-C photolysis was effective mainly for UV-susceptible compounds, particularly DCF and SMX, whereas CBZ and IBU showed limited removal under irradiation alone. Ozonation at pH ~6 markedly enhanced the apparent removal of ozone-reactive compounds, especially CBZ and DCF, and provided a near-neutral reference for evaluating the hybrid system. UV/O_3_ showed the most favourable overall performance under near-neutral conditions, particularly for CBZ and IBU, for which statistical analysis and SF values suggested apparent kinetic enhancement under combined UV/O_3_ conditions. In contrast, SMX showed a subadditive kinetic response, while DCF was excluded from the main SF assessment because its rapid decrease to concentrations approaching or falling below the lowest calibration level prevented robust kinetic fitting based only on quantifiable data.

The findings demonstrate that UV/O_3_ can improve parent-compound removal in multicomponent pharmaceutical systems, especially when the mixture contains compounds with low susceptibility to direct UV-C photolysis. Nevertheless, the investigated pharmaceutical concentrations were substantially higher than those typically detected in environmental waters and were selected to facilitate reliable kinetic assessment under controlled laboratory conditions. The observed improvement should be interpreted as apparent parent-compound removal under controlled model-water conditions rather than as evidence of mineralisation or complete elimination of transformation products. Accordingly, high parent-compound removal efficiencies should not be interpreted directly as evidence of reduced toxicity or environmental safety. The study is limited by the use of model water, the high COD associated with methanol introduced with the stock solution, the absence of dissolved-ozone and hydroxyl-radical monitoring, and the lack of transformation-product and toxicity assessment. Moreover, single ozonation was performed only at pH ~6; therefore, the pH-dependent conclusions apply to UV-C and UV/O_3_ treatments rather than to ozonation alone. Future work should include real wastewater matrices, ozone exposure and consumption measurements, radical-scavenging tests, LC-MS/MS identification of transformation products and complementary ecotoxicity assessment to determine whether rapid parent-compound removal is accompanied by reduced environmental risk.

## Figures and Tables

**Table 1:** Apparent pseudo-first-order kinetic parameters and synergy factors calculated for the UV/O_3_ process relative to individual UV-C and O_3_ treatments at pH ~6.

Compound	k_obs_ (min^−1^)			SF	Interpretation
	UV-C	O_3_	UV/O_3_		
SMX	0.0297	0.0340	0.0516	0.81	subadditive response
CBZ	0.0038	0.0774	0.1160	1.43	apparent synergy
IBU	0.0040	0.0204	0.0303	1.24	apparent synergy

## Data Availability

The original contributions presented in this study are included in the article/Supplementary Materials. Further inquiries can be directed to the corresponding author.

## Supplementary Materials

The following supporting information can be downloaded at: https://www.mdpi.com/article/10.3390/molecules31111930/s1, Table S1: Removal efficiency (%) for each analysed compound; Table S2: Apparent pseudo-first-order kinetic parameters for all compounds, processes and pH conditions; Table S3: Selected literature values of pseudo-first-order rate constants reported for UV-, O_3_^−^ and UV-assisted degradation of the investigated pharmaceuticals; Table S4: Tukey’s post hoc comparisons for UV-C, O_3_ and UV/O_3_ treatments at pH ~6 after 30 min; Table S5: Two-way ANOVA for UV-C and UV/O_3_ treatments across pH 3, ~6 and 8; Table S6: Tukey’s post hoc comparisons for UV-C and UV/O_3_ treatments across pH 3, ~6 and 8 after 30 min for SMX; Table S7: Tukey’s post hoc comparisons for UV-C and UV/O_3_ treatments across pH 3, ~6 and 8 after 30 min for CBZ; Table S8: Tukey’s post hoc comparisons for UV-C and UV/O_3_ treatments across pH 3, ~6 and 8 after 30 min for DCF; Table S9: Tukey’s post hoc comparisons for UV-C and UV/O_3_ treatments across pH 3, ~6 and 8 after 30 min for IBU. Refs. [59,60] are cited in Supplementary Materials.
