# Supplementary material for: Photo-Ozonation of Multiclass Pharmaceuticals in Model Water: Kinetic Comparison of UV-C, O3 and UV/O3 Under Selected pH Conditions
Source: Molecules. 2026 Jun 3;31(11):1930. doi: 10.3390/molecules31111930 (PMC13258770; doi:10.3390/molecules31111930)
Supplement: Supplementary file 1 [file molecules-31-01930-s001.zip › molecules-4333735-supplementary.pdf]

Supplementary Materials

Photo-Ozonation of Multiclass Pharmaceuticals in Model Water: Kinetic Comparison of UV-C, O_3 and UV/O_3 Under Selected pH Conditions

Klaudia Całus-Makowska, Anna Grosser * and Hanna Białek

Faculty of Infrastructure and Environment, Czestochowa University of Technology, J.H. Dąbrowskiego 69, 42-200 Częstochowa, Poland
* Correspondence: anna.grosser@pcz.pl

Table S1. Removal efficiency (%) for each analyzed compound

Removal efficiency	T [min]	Sulfamethoxazole (SMX)	Carbamazepine (CBZ)	Diclofenac (DCF)	Ibuprofen (IBU)
**UV-C pH 3**	30	79.98	11.24	91.24	45.62
	60	92.60	11.05	94.17	46.47
	90	96.22	11.57	100.00	46.58
	120	97.54	12.44	100.00	49.60
**UV-C pH 6**	30	91.29	30.92	97.83	17.24
	60	96.15	35.95	96.48	26.07
	90	96.57	38.16	96.19	33.20
	120	98.15	40.24	96.00	39.18
**UV-C pH 8**	30	48.38	12.35	80.53	7.25
	60	75.55	13.75	95.14	11.32
	90	88.96	17.89	98.31	15.31
	120	94.37	19.40	99.09	19.02
**Ozone pH 6**	30	41.35	67.19	87.26	75.94
	60	72.82	99.04	100.00	89.44
	90	90.25	100.00	100.00	90.47
	120	98.49	100.00	100.00	92.56
**UV/O_3 pH 3**	30	93.58	11.65	95.83	20.83
	60	98.61	30.69	96.03	30.88
	90	98.74	43.69	97.73	38.89
	120	98.62	55.63	100.00	44.95
**UV/O_3 pH 6**	30	95.49	96.92	100.00	85.68
	60	98.91	100.00	100.00	89.78
	90	99.08	100.00	100.00	94.41
	120	100.00	100.00	100.00	98.30
**UV/O_3 pH 8**	30	79.10	34.65	96.16	21.70
	60	96.23	41.95	96.78	37.01
	90	98.24	69.71	100.00	47.65
	120	98.09	87.93	100.00	55.12

Table S2. Apparent pseudo-first-order kinetic parameters for all compounds, processes and pH conditions

pH	Process	Compound	n_points	kobs_min^{-1}	SE_k	CI95_low	CI95_high	R^2	t1/2_min
**pH6**	O_3	SMX	5.0000	0.0340	0.0049	0.0183	0.0496	0.941	20.42
**pH6**	O_3	CBZ	3.0000	0.0774	0.0233	-0.2181	0.3729	0.917	8.95
**pH6**	O_3	DCF	2.0000	-	-	-	-	-	-
**pH6**	O_3	IBU	5.0000	0.0204	0.0053	0.0037	0.0371	0.834	33.97

**pH6**	UV-C	SMX	5.0000	0.0297	0.0082	0.0037	0.0557	0.815	23.33
**pH6**	UV-C	CBZ	5.0000	0.0038	0.0013	-0.0003	0.0079	0.741	182.32
**pH6**	UV-C	DCF	5.0000	0.0196	0.0151	-0.0284	0.0675	0.360	35.41
**pH6**	UV-C	IBU	5.0000	0.0040	0.0004	0.0029	0.0052	0.977	172.05
**pH6**	UV/O_3	SMX	4.0000	0.0516	0.0155	-0.0151	0.1183	0.847	13.44
**pH6**	UV/O_3	CBZ	2.0000	0.1160	-	-	-	-	5.98
**pH6**	UV/O_3	IBU	5.0000	0.0303	0.0048	0.0150	0.0456	0.930	22.87
**pH3**	UV-C	SMX	5.0000	0.0303	0.0044	0.0162	0.0443	0.940	22.91
**pH3**	UV-C	CBZ	5.0000	0.0009	0.0004	-0.0005	0.0023	0.592	772.04
**pH3**	UV-C	DCF	3.0000	0.0474	0.0195	-0.2005	0.2953	0.855	14.63
**pH3**	UV-C	IBU	5.0000	0.0046	0.0022	-0.0025	0.0117	0.588	149.80
**pH3**	UV/O_3	SMX	5.0000	0.0340	0.0118	-0.0035	0.0715	0.735	20.41
**pH3**	UV/O_3	CBZ	5.0000	0.0069	0.0004	0.0057	0.0082	0.990	100.18
**pH3**	UV/O_3	DCF	4.0000	0.0380	0.0163	-0.0319	0.1080	0.732	18.24
**pH3**	UV/O_3	IBU	5.0000	0.0048	0.0005	0.0033	0.0063	0.972	143.12
**pH8**	UV-C	SMX	5.0000	0.0243	0.0004	0.0230	0.0257	0.999	28.50
**pH8**	UV-C	CBZ	5.0000	0.0017	0.0004	0.0004	0.0029	0.859	418.86
**pH8**	UV-C	DCF	5.0000	0.0395	0.0039	0.0271	0.0518	0.972	17.56
**pH8**	UV-C	IBU	5.0000	0.0017	0.0001	0.0013	0.0021	0.987	405.52
**pH8**	UV/O_3	SMX	5.0000	0.0346	0.0072	0.0117	0.0576	0.885	20.01
**pH8**	UV/O_3	CBZ	5.0000	0.0150	0.0034	0.0041	0.0258	0.865	46.30
**pH8**	UV/O_3	DCF	3.0000	0.0573	0.0297	-0.3201	0.4346	0.788	12.11
**pH8**	UV/O_3	IBU	5.0000	0.0067	0.0003	0.0056	0.0078	0.992	103.71

Table S3. Selected literature values of pseudo-first-order rate constants reported for UV-, O_3- and UV-assisted degradation of the investigated pharmaceuticals

Compound	Process	Observed first-order rate constant (k)	Experimental conditions	Reference
**DCF**	UV-C LED photolysis	k = 1.39–3.07 min^{-1}; k' = 9.40 x 10^{-4} – 1.73 x 10^{-3} $mJ^{-1}cm^2$	DCF concentration: 20 mg L^{-1}; recirculating UV-LED photoreactor; direct photolysis at 265, 285 and 310 nm; irradiance from chemical actinometry: 21.98–31.18 mW cm^{-2}; pH not adjusted, measured at approximately 7.2; rate constants reported as time-based constants and UV-fluence-based constants.	[13]
**DCF**	Ozonation	k = 0.0742–0.0979 min^{-1}	Laboratory-scale bubble-column ozonation; DCF concentration: 50 mg dm^{-3}; pH-dependent ozonation evaluated in the range pH 5–9; pseudo-first-order degradation constants reported for different operating conditions; complete DCF removal achieved within approximately 6 min.	[55]
**CBZ**	UV-C photolysis	k_{obs} = 0.0676 min^{-1}	CBZ concentration: 0.042 mM; pH 5.6; aerobic conditions; UV-C irradiation at 254 nm; UV-C intensity: 12.61 mW cm^{-2}; reaction followed during prolonged irradiation.	[56]
**CBZ**	UV-C/TiO_2 photocatalysis	k_{obs} = 0.123 min^{-1}	CBZ concentration: 0.042 mM; TiO_2 dose: 0.5 g L^{-1}; pH 5.6; aerobic	[56]

			conditions; UV-C irradiation at 254 nm; UV-C intensity: 12.61 mW cm^{-2}.	
**CBZ**	UV-C/TiO_2 photocatalysis with radical scavenger	k_{obs} = 0.0029 min^{-1} with methanol; k_{obs} = 0.0696 min^{-1} without methanol	CBZ concentration: 0.042 mM; TiO_2 dose: 0.5 g L^{-1}; pH approximately 5.4–5.6; UV-C intensity: 3.65 mW cm^{-2}; methanol concentration: 50 mM; experiment used to evaluate the role of hydroxyl radicals during photocatalysis.	[56]
**IBU**	UV/chlorine AOP	k = 3.1 x 10^{-3}–5.5 x 10^{-4} s^{-1}equivalent to approximately 0.186–0.033 min^{-1}	IBU concentration: 50 µM; UV/chlorine treatment; pH range 6–9; pseudo-first-order kinetics reported; rate constant decreased as pH increased from 6 to 9.	[59]
**SMX**	UV_{254} photolysis	k = 0.170–0.932 min^{-1}	Low-pressure UV lamp at 254 nm; Milli-Q water and real sewage treatment plant effluent; SMX concentration: 1.0–10 mg L^{-1}; effects of initial concentration, pH, inorganic anions and humic acid evaluated; removal fitted to pseudo-first-order kinetics.	[58]
**SMX**	Ozonation	k = (0.9–9.8) x $10^{-3}$$s^{-1}$ equivalent to approximately 0.054–0.588\ min^{-1}	Aqueous SMX degradation by ozonation; operational variables included influent ozone gas concentration, initial SMX concentration, ionic strength, anions, humic acid and pH; degradation fitted to a pseudo-first-order kinetic model.	[60]
**SMX**	UV_{254} radiation	k = (1.7–18.9) x $10^{-3}$$s^{-1}$ equivalent to approximately 0.102–1.134 min^{-1}	UV radiation at 254 nm using low-pressure mercury lamps; operational variables included UV light intensity, initial SMX concentration, pH, ionic strength, anions and humic acid; degradation fitted to a pseudo-first-order kinetic model.	[60]

Table S4. Tukey's post hoc comparisons for UV-C, O_3 and UV/O_3 treatments at pH ~6 after 30 min

Compound	Group 1	Group 2	Mean difference (%)	95% CI	p-adjusted	Significant
**SMX**	O_3	UV-C	49.95	39.94 to 59.96	$p < 0.001$	Yes
**SMX**	O_3	UV/O_3	54.15	44.14 to 64.16	$p < 0.001$	Yes
**SMX**	UV-C	UV/O_3	4.20	−5.81 to 14.21	$p = 0.451$	No
**CBZ**	O_3	UV-C	−36.28	−45.10 to −27.46	$p < 0.001$	Yes
**CBZ**	O_3	UV/O_3	29.73	20.91 to 38.55	$p < 0.001$	Yes
**CBZ**	UV-C	UV/O_3	66.01	57.19 to 74.82	$p < 0.001$	Yes
**DCF**	O_3	UV-C	10.57	1.09 to 20.05	$p = 0.033$	Yes
**DCF**	O_3	UV/O_3	12.74	3.26 to 22.22	$p = 0.015$	Yes
**DCF**	UV-C	UV/O_3	2.17	−7.31 to 11.65	$p = 0.772$	No
**IBU**	O_3	UV-C	−58.69	−67.07 to −50.32	$p < 0.001$	Yes
**IBU**	O_3	UV/O_3	9.74	1.37 to 18.11	$p = 0.027$	Yes
**IBU**	UV-C	UV/O_3	68.43	60.06 to 76.81	$p < 0.001$	Yes

Table S5. Two-way ANOVA for UV-C and UV/O_3 treatments across pH 3, ~6 and 8

Compound	Source	F(df)	p-value	Partial η^2
**SMX**	Process	$F(1,12) = 68.56$	$p < 0.001$	0.851
**SMX**	pH	$F(2,12) = 84.67$	$p < 0.001$	0.934
**SMX**	Process × pH	$F(2,12) = 15.80$	$p < 0.001$	0.725
**CBZ**	Process	$F(1,12) = 789.29$	$p < 0.001$	0.985

**CBZ**	pH	F(2,12) = 909.18	p < 0.001	0.993
**CBZ**	Process × pH	F(2,12) = 335.61	p < 0.001	0.982
**DCF**	Process	F(1,12) = 14.06	p = 0.003	0.539
**DCF**	pH	F(2,12) = 9.39	p = 0.004	0.610
**DCF**	Process × pH	F(2,12) = 4.33	p = 0.038	0.419
**IBU**	Process	F(1,12) = 379.30	p < 0.001	0.969
**IBU**	pH	F(2,12) = 461.27	p < 0.001	0.987
**IBU**	Process × pH	F(2,12) = 738.80	p < 0.001	0.992

Table S6. Tukey's post hoc comparisons for UV-C and UV/O_3 treatments across pH 3, ~6 and 8 after 30 min for SMX

Group 1	Group 2	Mean difference (%)	95% CI	p-adjusted	Significant
**UV/O_3 pH 3**	UV/O_3 pH ~6	1.91	−9.45 to 13.28	p = 0.992	No
**UV/O_3 pH 3**	UV/O_3 pH 8	−14.48	−25.84 to −3.11	p = 0.011	Yes
**UV/O_3 pH 3**	UV pH 3	−13.60	−24.96 to −2.24	p = 0.016	Yes
**UV/O_3 pH 3**	UV pH ~6	−2.29	−13.65 to 9.08	p = 0.981	No
**UV/O_3 pH 3**	UV pH 8	−45.20	−56.56 to −33.83	p < 0.001	Yes
**UV/O_3 pH ~6**	UV/O_3 pH 8	−16.39	−27.76 to −5.03	p = 0.004	Yes
**UV/O_3 pH ~6**	UV pH 3	−15.51	−26.88 to −4.15	p = 0.006	Yes
**UV/O_3 pH ~6**	UV pH ~6	−4.20	−15.56 to 7.16	p = 0.809	No
**UV/O_3 pH ~6**	UV pH 8	−47.11	−58.48 to −35.75	p < 0.001	Yes
**UV/O_3 pH 8**	UV pH 3	0.88	−10.49 to 12.24	p = 1.000	No
**UV/O_3 pH 8**	UV pH ~6	12.19	0.83 to 23.55	p = 0.033	Yes
**UV/O_3 pH 8**	UV pH 8	−30.72	−42.08 to −19.36	p < 0.001	Yes
**UV pH 3**	UV pH ~6	11.31	−0.05 to 22.68	p = 0.051	No
**UV pH 3**	UV pH 8	−31.60	−42.96 to −20.23	p < 0.001	Yes
**UV pH ~6**	UV pH 8	−42.91	−54.28 to −31.55	p < 0.001	Yes

Table S7. Tukey's post hoc comparisons for UV-C and UV/O_3 treatments across pH 3, ~6 and 8 after 30 min for CBZ

Group 1	Group 2	Mean difference (%)	95% CI	p-adjusted	Significant
**UV/O_3 pH 3**	UV/O_3 pH ~6	85.27	79.15 to 91.40	p < 0.001	Yes
**UV/O_3 pH 3**	UV/O_3 pH 8	23.00	16.87 to 29.12	p < 0.001	Yes
**UV/O_3 pH 3**	UV-C pH 3	−0.41	−6.54 to 5.71	p = 1.000	No
**UV/O_3 pH 3**	UV-C pH ~6	19.27	13.14 to 25.39	p < 0.001	Yes
**UV/O_3 pH 3**	UV-C pH 8	0.71	−5.42 to 6.83	p = 0.999	No
**UV/O_3 pH ~6**	UV/O_3 pH 8	−62.27	−68.40 to −56.15	p < 0.001	Yes
**UV/O_3 pH ~6**	UV-C pH 3	−85.68	−91.81 to −79.56	p < 0.001	Yes
**UV/O_3 pH ~6**	UV-C pH ~6	−66.01	−72.13 to −59.88	p < 0.001	Yes
**UV/O_3 pH ~6**	UV-C pH 8	−84.57	−90.69 to −78.44	p < 0.001	Yes
**UV/O_3 pH 8**	UV-C pH 3	−23.41	−29.53 to −17.29	p < 0.001	Yes
**UV/O_3 pH 8**	UV-C pH ~6	−3.73	−9.86 to 2.39	p = 0.373	No
**UV/O_3 pH 8**	UV-C pH 8	−22.29	−28.42 to −16.17	p < 0.001	Yes
**UV-C pH 3**	UV-C pH ~6	19.68	13.55 to 25.80	p < 0.001	Yes
**UV-C pH 3**	UV-C pH 8	1.12	−5.01 to 7.24	p = 0.988	No
**UV-C pH ~6**	UV-C pH 8	−18.56	−24.68 to −12.44	p < 0.001	Yes

Table S8. Tukey's post hoc comparisons for UV-C and UV/O_3 treatments across pH 3, ~6 and 8 after 30 min for DCF

Group 1	Group 2	Mean difference (%)	95% CI	p-adjusted	Significant
**UV/O_3 pH 3**	UV/O_3 pH ~6	4.17	−7.42 to 15.75	p = 0.825	No
**UV/O_3 pH 3**	UV/O_3 pH 8	0.33	−11.25 to 11.91	p = 1.000	No
**UV/O_3 pH 3**	UV-C pH 3	−4.59	−16.18 to 6.99	p = 0.764	No
**UV/O_3 pH 3**	UV-C pH ~6	2.00	−9.58 to 13.58	p = 0.991	No
**UV/O_3 pH 3**	UV-C pH 8	−15.30	−26.89 to −3.72	p = 0.008	Yes
**UV/O_3 pH ~6**	UV/O_3 pH 8	−3.84	−15.42 to 7.75	p = 0.867	No
**UV/O_3 pH ~6**	UV-C pH 3	−8.76	−20.34 to 2.83	p = 0.187	No
**UV/O_3 pH ~6**	UV-C pH ~6	−2.17	−13.75 to 9.42	p = 0.987	No
**UV/O_3 pH ~6**	UV-C pH 8	−19.47	−31.05 to −7.89	p = 0.001	Yes
**UV/O_3 pH 8**	UV-C pH 3	−4.92	−16.51 to 6.66	p = 0.712	No
**UV/O_3 pH 8**	UV-C pH ~6	1.67	−9.92 to 13.25	p = 0.996	No
**UV/O_3 pH 8**	UV-C pH 8	−15.63	−27.22 to −4.05	p = 0.007	Yes
**UV-C pH 3**	UV-C pH ~6	6.59	−4.99 to 18.17	p = 0.440	No
**UV-C pH 3**	UV-C pH 8	−10.71	−22.30 to 0.87	p = 0.076	No
**UV-C pH ~6**	UV-C pH 8	−17.30	−28.89 to −5.72	p = 0.003	Yes

Table S9. Tukey's post hoc comparisons for UV-C and UV/O_3 treatments across pH 3, ~6 and 8 after 30 min for IBU

Group 1	Group 2	Mean difference (%)	95% CI	p-adjusted	Significant
**UV/O_3 pH 3**	UV/O_3 pH ~6	64.85	59.07 to 70.64	p < 0.001	Yes
**UV/O_3 pH 3**	UV/O_3 pH 8	0.88	−4.91 to 6.66	p = 0.995	No
**UV/O_3 pH 3**	UV-C pH 3	24.80	19.01 to 30.58	p < 0.001	Yes
**UV/O_3 pH 3**	UV-C pH ~6	−3.58	−9.37 to 2.20	p = 0.357	No
**UV/O_3 pH 3**	UV-C pH 8	−13.58	−19.36 to −7.79	p < 0.001	Yes
**UV/O_3 pH ~6**	UV/O_3 pH 8	−63.98	−69.76 to −58.19	p < 0.001	Yes
**UV/O_3 pH ~6**	UV-C pH 3	−40.06	−45.84 to −34.27	p < 0.001	Yes
**UV/O_3 pH ~6**	UV-C pH ~6	−68.43	−74.22 to −62.65	p < 0.001	Yes
**UV/O_3 pH ~6**	UV-C pH 8	−78.43	−84.21 to −72.65	p < 0.001	Yes
**UV/O_3 pH 8**	UV-C pH 3	23.92	18.14 to 29.71	p < 0.001	Yes
**UV/O_3 pH 8**	UV-C pH ~6	−4.46	−10.24 to 1.33	p = 0.174	No
**UV/O_3 pH 8**	UV-C pH 8	−14.45	−20.24 to −8.67	p < 0.001	Yes
**UV-C pH 3**	UV-C pH ~6	−28.38	−34.16 to −22.59	p < 0.001	Yes
**UV-C pH 3**	UV-C pH 8	−38.37	−44.16 to −32.59	p < 0.001	Yes
**UV-C pH ~6**	UV-C pH 8	−10.00	−15.78 to −4.21	p = 0.001	Yes
